# Optimized Protoplasts Preparation of Fungus *Magnaporthe oryzae* by Novel Myxolase F Lyase

**DOI:** 10.3390/jof12100709

**Published:** 2026-09-22

**Authors:** Jun Liu, Mingze Gao, Erya Tang, Fanghui Ding, Zhoukun Li, Zhongli Cui

**Affiliations:** 1 Key Laboratory of Agricultural Environmental Microbiology, Ministry of Agriculture and Rural Affairs, College of Life Sciences, Nanjing Agricultural University, Nanjing 210095, China; 2 Nanjing Foreign Language School, Nanjing 210008, China; 3 Nanjing Myxobiotech Biotechnological Co., Ltd., Nanjing 210014, China

**Keywords:** fungal cell wall, decomposing enzyme, regeneration, transformation

## Abstract

Rice blast disease is one of most devastating fungal diseases caused by *Magnaporthe oryzae*. The preparation and transformation of protoplasm are of vital importance for understanding the pathogenic mechanism of *M. oryzae* and achieving the control of rice blast disease. In this study, we aim to optimize conditions for preparing and regenerating the protoplasts of *M. oryzae* using a novel commercial fungal cell wall-decomposing enzyme, Myxolase F lyase. By optimizing the enzyme concentration, lysis time and pH, osmatic stabilizer and agar percentage in regeneration medium, an applicable protoplast preparation system for *M. oryzae* was established. Following lysis of *M. oryzae* mycelium with 5 mg/mL Myxolase F lyase for 3 h, a 49.7% regeneration rate was achieved using a regenerative medium consisting of 0.8 M sucrose and 1% agar at pH 7.0 (50 mM Tris-HCl buffer) under optimum conditions. Moreover, the prepared *M. oryzae* protoplasts were available for subsequent genetic transformation, with a regeneration rate of 54.28% using Myxolase F compared with 26.86% using the commercial enzyme. The simplified operations, robust reproducibility and improved efficiency give Myxolase F lyase an advantage over other available products, thus providing powerful tools for the efficient genetic transformation of *M. oryzae*.

## 1. Introduction

Rice blast disease, caused by the fungal pathogen *Magnaporthe oryzae*, is one of the most destructive diseases affecting rice cultivation worldwide and has been reported to cause 70–80% yield loss [1]. The rice blast fungus *M. oryzae* exhibits remarkable ecological adaptability, allowing it to infect rice plants at nearly all growth stages [2]. Understanding the pathogenic mechanisms of *M. oryzae* is important for developing effective strategies to control rice blast disease [3]. However, *M. oryzae* populations exhibit high genetic diversity and diverse pathotypes, and their interactions with different rice cultivars result in a complex molecular basis of pathogenicity [4,5,6]. Therefore, an efficient and stable genetic manipulation system is required for functional characterization of pathogenicity-related genes and for dissecting the infection mechanisms of *M. oryzae*.

Fungal protoplasts are important experimental materials for physiological and genetic studies [7]. Efficient genetic transformation requires exogenous DNA to pass through the fungal cell wall and enter the cell. However, the fungal cell wall is essential for fungal growth and survival. In most fungi, the inner cell wall skeleton is mainly composed of chitin and β-glucans, which form a flexible viscoelastic framework [8]. In *M. oryzae*, α-1,3-glucan spatially and functionally masks β-1,3-glucan and chitin in the cell wall of infectious hyphae, which could reduce the accessibility of cell wall-degrading enzymes and increase resistance to enzymatic digestion [9]. In addition, fungal cell wall hydrolysis by cell wall-degrading enzymes could trigger the cell wall integrity (CWI) pathway that is primarily responsible for maintaining cell wall homeostasis. In the enzyme complex, only β-1,3-glucanase and protease activities are essential to elicit Slt2 activation [10]. The signal is transduced through a composite high-osmolarity glycerol (HOG) pathway and the CWI pathway [11]. Activation of the CWI signaling pathway may have an impact on pathogenicity. Therefore, efficient fungal cell wall-degrading enzymes and an appropriate preparation process are necessary for protoplast preparation in *M. oryzae*.

Protoplast-mediated transformation is one of the most widely used approaches for fungal genome manipulation, and the quality of protoplasts directly affects the efficiency of downstream genetic operations, including gene replacement, gene knockout, and functional characterization [12,13,14]. Previous studies on *M. oryzae* protoplast preparation have mainly focused on screening suitable lytic enzymes and optimizing experimental conditions, such as enzyme combinations and mycelial status [15]. Similar optimization strategies, including enzyme selection, osmotic stabilizers, digestion conditions, and regeneration media, have also been widely applied in other fungi [16,17]. Although these studies have improved protoplast yield and regeneration efficiency, relying on enzyme cocktails and complicated operational steps may increase experimental cost and reduce reproducibility. Therefore, establishing a simple, efficient, and reproducible protoplast preparation system remains important for genetic transformation and functional genomic studies in *M. oryzae*. Myxobacteria are known for their ability to decompose complex biomass types by various carbohydrate-active enzymes [18] and are thereby regarded as novel versatile cell factories [19]. Meantime, myxobacteria are facultative predators possessing the capability to feed on a broad range of soil microorganisms by producing abundant cell wall-decomposing enzymes [20,21,22], raising the possibility of the preparation of fungal protoplasts by enzyme cocktail composed of chitinases and β-glucanases. The objective of this study was to establish a genetic transformation system for *M. oryzae* using a commercial lytic, Myxolase F. We hypothesized that Myxolase F may possess efficient ability to decompose fungal cell wall compared to other commercial enzymes due its optimal enzyme-cocktail ratio, and, therefore have potential application in protoplast preparation. To assess the potential of Myxolase F as a genetic tool, we thoroughly examined the preparation parameters and conversion efficiency.

## 2. Materials and Methods

### 2.1. Strains, Growth Conditions and Reagents

*M. oryzae* Guy11 has been widely used as wild-type strain for the investigation of plant-microbe-pathogen interactions and was used as a model fungus establish a genetic system in this study. The conditions for the growth of *M. oryzae* were the same as those published earlier [23]. Complete medium (CM) or PDA medium was used for the vegetative growth of the *M. oryzae* strain when necessary [24]. MyxolaseF was provided by MyxoBiotech Co., Ltd., Company (Lot. No. 251115A, Nanjing, China). Unless otherwise stated, all other chemicals were of analytical grade. The components of the media and solutions used for protoplast preparation and regeneration in this study are provided in Table 1. The formula of the buffer used to adjust the pH in TB3 media and osmotic stabilizers are provided in Table 2. Liquid CM medium was used to collect the mycelium of *M. oryzae*. Agar discs (0.1 × 0.1 cm) were removed from the edge of actively growing *M. oryzae* and transferred to liquid CM medium and incubated at 28 °C for 48 h when necessary.

### 2.2. Optimization of Enzymatic Lysis Conditions for Protoplast Preparation of M. oryzae

Different concentrations of Myxolase F were prepared to evaluate their effects on protoplast release of *M. oryzae*. Considering the insufficient mycelial decomposition caused by low concentration of Myxolase F, 0.05, 0.1, 0.15, 0.2, 0.25, and 0.3 g of Myxolase F (dry weight) were weighted and dissolved in 20 mL of 0.7 M NaCl, producing the corresponding lytic solution with final concentrations of 2.5, 5.0, 7.5, 10.0, 12.5, and 15.0 mg/mL, respectively. The enzyme solutions were used for enzymatic lysis of prepared mycelium of *M. oryzae*. To investigate the optimal reaction time, the test digestion time was set to 1, 2, 3, and 4 h, respectively, and the amount of protoplasm released was quantified by microscopic counting.

Several factors affecting Myxolase F-mediated protoplast preparation were further examined. *M. oryzae* was cultured in liquid CM medium for 24, 48, 60, and 72 h, and the corresponding mycelium was collected with the aim of comparing the effect of mycelial age on protoplast yield. Enzymatic reactions were carried out at 20, 25, 30, and 35 °C to assess the influence of temperature on protoplast preparation. The effect of shaking speed was evaluated by incubating the reaction mixtures at 60, 80, 100, and 120 rpm. Six osmotic stabilizers, including KCl, NaCl, MgSO_4_, mannitol, sorbitol, and sucrose, were compared for their ability to maintain protoplast stability during enzymatic lysis. Otherwise, the concentration of the osmotic stabilizer was adjusted from 0.3 M to 0.7 M to explore its effect on protoplast preparation.

Protoplast yield was expressed as

*Yp* = *NV*/*M*

where *Yp* represents the protoplast yield, *N* represents the protoplast concentration (cells/mL), *V* represents the volume of enzymatic digestion solution (mL), and *M* represents the wet weight of fresh mycelia (g).

### 2.3. Protoplast Regeneration

Protoplasts of *M. oryzae* were prepared under the optimized enzymatic lysis conditions. Undigested mycelial fragments were removed by filtration through three layers of lens wipes. Protoplasts were collected by centrifugation at 3000× *g* for 5 min. The collected protoplasts were washed once with 0.7 M NaCl and twice with STC buffer, followed by centrifugation at 3000× *g* for 5 min. The protoplast pellet was resuspended in 20 mL STC buffer, and the number of protoplasts in suspension was counted using a hemocytometer with optimal dilution. Then, 100 μL of the diluted protoplast suspension was mixed with 20 mL of TB3 medium and poured into Petri dishes. All the comparative experiments were performed with consistent dilution and initial protoplast concentration. The plates were incubated at 26 °C for 3 days, after which the regeneration rate was calculated by colony counting.

### 2.4. Optimization of Protoplast Regeneration Conditions

To determine the effects of different osmotic stabilizers on protoplast regeneration, TB3 regeneration medium was supplemented with mannitol, sorbitol, or sucrose with different concentrations (0.6, 0.7, 0.8, 0.9, and 1.0 M). To determine the optimal pH for protoplast regeneration, TB3 medium was adjusted to different pH values using the buffers listed in Table 2. The final concentrations of agar were adjusted to 0.7, 0.8, 0.9, 1.0, 1.1, and 1.2% with the aim to evaluate their effects on protoplast regeneration. In addition, under the optimal conditions, commercialized enzyme (Lysing enzyme, 2028-12-24, 1:100 in sodium chloride solution, Nanjing, China) was used to compare with Myxolase F, to compare their efficiency in protoplast preparation and regeneration.

### 2.5. PEG-Mediated Transformation of M. oryzae Protoplasts

For determining the PEG-mediated transformation, freshly prepared *M. oryzae* protoplasts were resuspended in STC buffer with a final concentration of approximately 1 × 10^6^ protoplasts/mL. An amount of 150 μL of protoplast suspension was transferred into a sterile centrifuge tube. Then, 3–5 μg of circular pDL2 plasmid carrying GFP and a hygromycin B was added to the suspension and gently mixed. Subsequently, 160 μL of STC buffer was added and mixed gently, followed by the addition of 40 μL SPTC solution. The mixture was incubated on ice for 2 h with gentle inversion every 15 min. After incubation, 1 mL of SPTC solution was added to the transformation mixture and gently mixed, followed by incubation at room temperature for 20 min. Then, 8 mL of antibiotic-free TB3 liquid medium was added, and the mixture was incubated at 28 °C in the dark for 3 h for cell recovery. The recovered transformation mixture was mixed with 60 mL molten TB3 solid medium that cooled to approximately 45–50 °C, and then poured into 90 mm Petri dishes to form the first layer.

The plate was placed at room temperature to air dry, and then 10 mL TB3 of solid medium containing 100 μg/mL of hygromycin B was poured onto the first layer, which was regarded as the selective overlay. The plates were air-dried and incubated at 28 °C in the dark for 5–7 days. Putative transformants growing on hygromycin B-containing plates were picked and transferred to fresh PDA medium containing 100 μg/mL hygromycin B for selective growth. Stable transformants were further examined by GFP fluorescence observation and PCR amplification.

### 2.6. Statistical Analysis and Reproducibility

All experiments were repeated at least three times where similar results were found. Data represent mean ± SE of all biological replicates. Statistical significance was assessed by a one-way analysis of variance (ANOVA) followed by Duncan’s test as a multiple-comparison test using SPSS 20.0 (IBM Inc., Chicago, IL, USA). All results were representative of at least three independent experiments with similar outcomes.

## 3. Results

### 3.1. Myxolase F Efficiently Released Protoplasts from Hydrolysis of M. oryzae Mycelium

Myxolase F from myxobacteria possesses the ability to decompose the fungal cell wall. We hypothesized that Myxolase F could break down the cell wall and release fungal protoplasts. Compared to normal *M. oryzae* mycelium (Figure 1A), Myxolase F treatment produced numerous spherical protoplasts as observed under the microscope (Figure 1B). While the cell wall integrity of *M. oryzae* remained intact without treatment, decomposed structures were observed after treatment with Myxolase F, and most released protoplasts were round and intact. These results indicate that Myxolase F could effectively degrade the cell wall of *M. oryzae* and release protoplasts with intact membrane structures. The obtained protoplasts were subsequently used for yield determination, regeneration assays, and PEG-mediated transformation.

### 3.2. Effects of Myxolase F Concentration and Digestion Time on Protoplast Yield

Enzyme dosage and hydrolytic time are important for the complete decomposition of the fungal cell wall during protoplast preparation. Hence, the effects of Myxolase F concentration and digestion time on the yield of *M. oryzae* protoplasts were investigated. As shown in Figure 2A, treatment of *M. oryzae* mycelium with 5.0 mg/mL Myxolase F resulted in the highest protoplast yield, reaching 6.61 × 10^8^/g FW. A low concentration of Myxolase F failed to completely decompose the *M. oryzae* cell wall, while a high concentration led to the rapid decomposition of the mycelium, preventing the timely release of the protoplasts. Therefore, 5.0 mg/mL was selected as the optimal enzyme concentration. Under the optimal enzyme concentration, the highest number of protoplast formation was observed as 8.00 × 10^8^/g FW after 3 h treatment (Figure 2B). Thus, 3 h of hydrolysis with Myxolase F was selected as the optimal time for subsequent experiments.

### 3.3. Optimization of Enzymatic Lysis Conditions for M. oryzae Protoplast Preparation

The cultivation time of the mycelium markedly affected protoplast yield. As shown in Figure 3A, the highest yield was obtained from mycelia cultured for 48 h, reaching 5.28 × 10^8^/g FW. As the cultivation time increased, protoplast yield gradually decreased, mainly because of changes in the quality of the mycelium during long-time culture. Therefore, mycelium after 48 h of incubation was used for subsequent experiments. Reaction temperature and shaking speed also influenced Myxolase F-mediated cell wall lysis. As shown in Figure 3B, the highest protoplast yield of 5.17 × 10^8^/g FW was obtained at 30 °C. Among the tested shaking speeds, treatment with 80 rpm produced the highest protoplast yield of 4.88 × 10^8^/g FW, whereas higher shaking speeds reduced protoplast production (Figure 3C).

Since protoplast yield is the overall result of the interactions among many factors, the influence of the osmotic stabilizer on protoplast preparation was also investigated. Among the six tested osmotic stabilizers, sucrose produced the highest protoplast yield (Figure 3D), in contrast to previous findings that inorganic salts were optimal osmotic pressure stabilizers for protoplast preparation. Further optimization showed that 0.4 M sucrose was the most suitable concentration, with a protoplast yield of 5.46 × 10^8^/g FW (Figure 3E). The pH of the enzyme solution also affected protoplast production, and the highest yield was obtained at pH 6.2 (Figure 3F). Therefore, the optimal conditions for protoplast preparation using Myxolase F were determined to be mycelium cultured for 48 h and digested with 5.0 mg/mL Myxolase F for 3 h at 30 °C and 80 rpm, using 0.4 M sucrose as the osmotic stabilizer and a reaction pH value of 6.2.

### 3.4. Optimization of Protoplast Regeneration Conditions

The regeneration efficiency of *M. oryzae* protoplasts was affected by multiple factors, including osmotic stabilizer, medium pH, and agar concentration. As shown in Figure 4A, sucrose was an excellent osmotic stabilizer for promoting protoplast regeneration, compared to mannitol and sorbitol. Treatment with 0.8 M sucrose resulted in a 49.95% regeneration rate, and this concentration was selected as the optimal concentration of the osmotic stabilizer for protoplast regeneration. The pH of the TB3 medium also influenced protoplast regeneration. The highest regeneration rate was observed at pH 7.0 when Tris-HCl was used as the buffer (Figure 4B), whereas other pH values reduced regeneration efficiency, possible owing to the acid sensitivity of the plasma membrane. Agar concentration further affected colony recovery from protoplasts, and 1.0% agar produced the highest regeneration rate among the tested concentrations (Figure 4C). Taken together, TB3 medium containing 0.8 M sucrose and 1.0% agar at pH 7.0 was selected as the optimal medium for *M. oryzae* protoplast regeneration.

### 3.5. Comparison of Myxolase F with a Commercial Control Enzyme in Protoplasts Preparation

The efficiency of Myxolase F was compared with that of a commercial control enzyme under the same experimental conditions and at the optimally recommended dosage. As shown in Figure 5A, Myxolase F produced a higher protoplast yield than the commercial lysing enzyme commonly used for *M. oryzae* protoplast preparation, reaching approximately 6.3 × 10^8^/g FW. In addition, protoplasts prepared with Myxolase F showed a markedly higher regeneration rate than those prepared with the commercial enzyme. The regeneration rate reached 54.28% in the Myxolase F group, compared with the 26.86% regeneration rate obtained in the commercial enzyme group (Figure 5B). These results indicate that Myxolase F, as a novel commercial enzyme cocktail, is effective for preparing regenerable *M. oryzae* protoplasts.

### 3.6. Genetic Transformation in M. oryzae Protoplasts Prepared by Myxolase F

The protoplasts prepared using Myxolase F were further tested for PEG-mediated transformation with the circular pDL2 plasmid carrying GFP and hygromycin B as a resistance marker. After selection on TB3 agar medium containing 100 μg/mL hygromycin B, growth of resistant transformants was observed. Under the optimized transformation conditions, an average of 156 transformants/μg DNA was obtained over 10 replicates (Figure 6A). The selected transformants were able to grow on hygromycin B-containing agar medium (Figure 6B) with normal mycelial morphology (Figure 6C), and clear GFP fluorescence was observed in the mycelia under fluorescence microscopy compared to the control (Figure 6D), indicating efficient genetic transformation of the *M. oryzae* protoplasts. These results indicate that Myxolase F-prepared protoplasts are suitable for PEG-mediated genetic transformation in *M. oryzae*.

## 4. Discussion

Protoplast quality and quantity are critical factors for successful genetic transformations and functional genomic research. Investigations of the pathogenicity of fungal pathogens commonly engaged in fungal genetics involve gene manipulation procedures based on protoplasts [25]. Rice blast disease caused by *M. oryzae* is a major constraint on worldwide rice production, understanding the biology of plant infection is a priority for the development of new disease control strategies [26]. In the *M. oryzae* cell wall, chitin, β-1,3-glucan, and α-1,3-glucan are robust skeletal polysaccharides that impart mechanical strength. Chitin is the most rigid polysaccharide and is cross-linked with β-1,3-glucan, whereas α-1,3-glucan spatially and functionally masks β-1,3-glucan and chitin, thereby forming a flexible viscoelastic framework [8]. These structural features could affect the accessibility of cell wall-degrading enzymes. Hence, protoplast preparation in fungi relies on multiple enzyme combinations to decompose the cell wall, including enzyme cocktails such as Driselase, Lysozyme, and Snailase. Myxolase F is a novel commercial fungal cell wall lytic enzyme derived from predatory myxobacteria [18,19] and is an enzyme cocktail composed of multiple cell wall lytic enzymes, including chitinase, β-1,3-glucanase, β-1,6-glucanase, and protease. Compared with conventional protoplast preparation methods that often rely on combination of multiple enzymes, the use of Myxolase F provides a simpler enzymatic system for fungal cell wall degradation. In this study, Myxolase F was successfully applied to *M. oryzae* protoplast preparation, producing large numbers of spherical and intact protoplasts suitable for regeneration and PEG-mediated transformation.

Optimization of protoplast preparation conditions in *Pseudozyma flocculosa* [27], *Antrodia cinnamomea* [28], *Eutypella* [16], *Lyophyllum decastes* [29], *Sclerotiophoma versabilis* [30], and *Cordyceps cicadae* [31], has been intensely investigated by considering the incubation time, digestion time, the type of protective agent, and enzyme concentration. However, few studies have been conducted on the development of solubilizing enzymes and the construction of protoplast preparation systems. It was found that the highest protoplast yield in fungi was obtained with a combination of Driselase and lysing enzymes [30,32]. In addition, Novozym 234 has been reported to be active against a wide range of fungal species [33]. Myxolase F was commercially developed from predatory myxobacteria owing to their ability to prey on fungi using complex CWDEs, and the single-component treatment avoids the complex operational process associated with multiple enzyme combinations. Myxolase F is considered to be a better choice based on cost-effectiveness. However, considering the difficulty of cross-study comparisons, we are unable to compare the preparation efficiency of different fungal protoplasts using Myxolase F and other commercial enzymes under consistent conditions, an issue that should be considered in the future.

The success of protoplast isolation and regeneration depends on various factors, including the age of mycelia, lytic enzyme concentration, osmotic stabilizer, temperature, pH, and incubation time [16]. The optimal lysis condition was digestion of 48-h mycelia with 5.0 mg/mL Myxolase F for 3 h at 30 °C and 80 rpm, using 0.4 M sucrose as the osmotic stabilizer at pH 6.2. These results indicate that both the physiological status of mycelia and the enzymatic reaction environment are critical for efficient protoplast release. This is consistent with previous studies showing that fungal protoplast preparation is highly dependent on enzyme type, digestion conditions, and mycelial status [13,16]. It should be pointed out that the decrease in protoplast production at 4 h may be due in part to the destruction of the integrity of the cell membrane caused by the long incubation time and the adverse effects on the physiological activities of protoplasts [34]. This could be verified by using the viability of protoplasts as an indicator during the optimization process for each parameter. The major advantage of Myxolase F is its ability to generate protoplasts with high regeneration capacity and efficient genetic transformation. TB3 medium containing 0.8 M sucrose and 1.0% agar at pH 7.0 supported efficient regeneration of *M. oryzae* protoplasts. Moreover, compared with the commercial control enzyme, Myxolase F produced a higher protoplast yield and regeneration rate under the same experimental conditions. These results suggest that Myxolase F not only promotes cell wall degradation but also helps maintain protoplast viability. The use of a single commercial enzyme may also reduce operational complexity and improve reproducibility compared with traditional enzyme cocktails. Moreover, apart from protoplast preparation and regeneration, protoplast vitality should be assessed using active staining; this is valuable for better utilization and optimization of this system in future research. In addition, the analysis of differences among various processing parameters still requires further controls to establish a stable preparation system [35].

In summary, these results indicate that Myxolase F is an effective fungal cell wall lytic enzyme for *M. oryzae* protoplast preparation. Myxolase F showed high lytic efficiency at a relatively low concentration and generated protoplasts with excellent regeneration capacity. Moreover, Myxolase F could be used alone, avoiding multiple enzyme cocktails and simplifying the protoplast preparation procedure. Through systematic optimization of enzymatic lysis and regeneration conditions, this study established an efficient, reproducible, and robust workflow for *M. oryzae* protoplast preparation, regeneration, and PEG-mediated transformation, supporting future gene function and pathogenicity-related studies in the rice blast fungus.

## Figures and Tables

**Figure 1:**
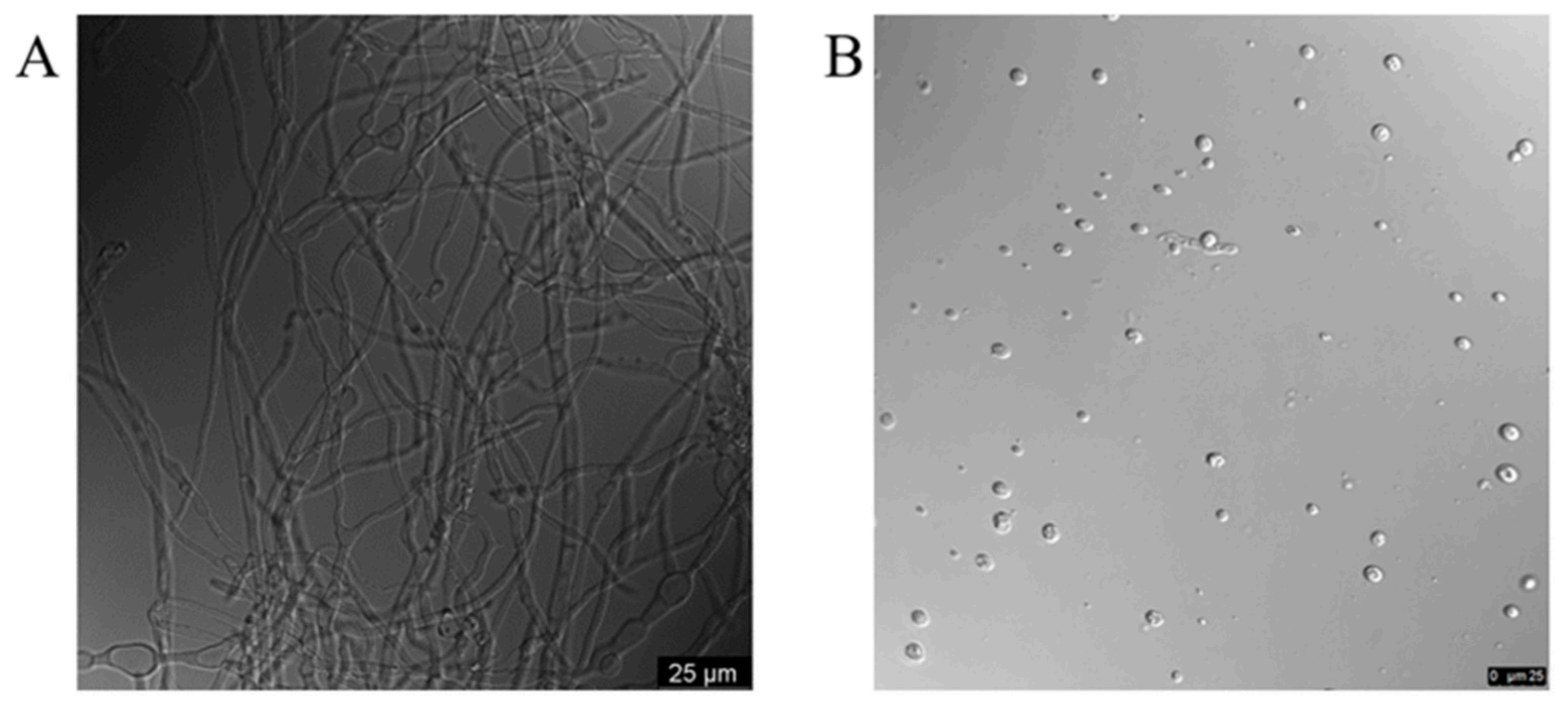
Microscopic observation of *M. oryzae* protoplasts prepared using commercial Myxolase F. (**A**) Normal *M. oryzae* mycelium without enzymatic treatment. (**B**) Spherical protoplasts prepared using Myxolase F. Scale bars: 25 μm.

**Figure 2:**
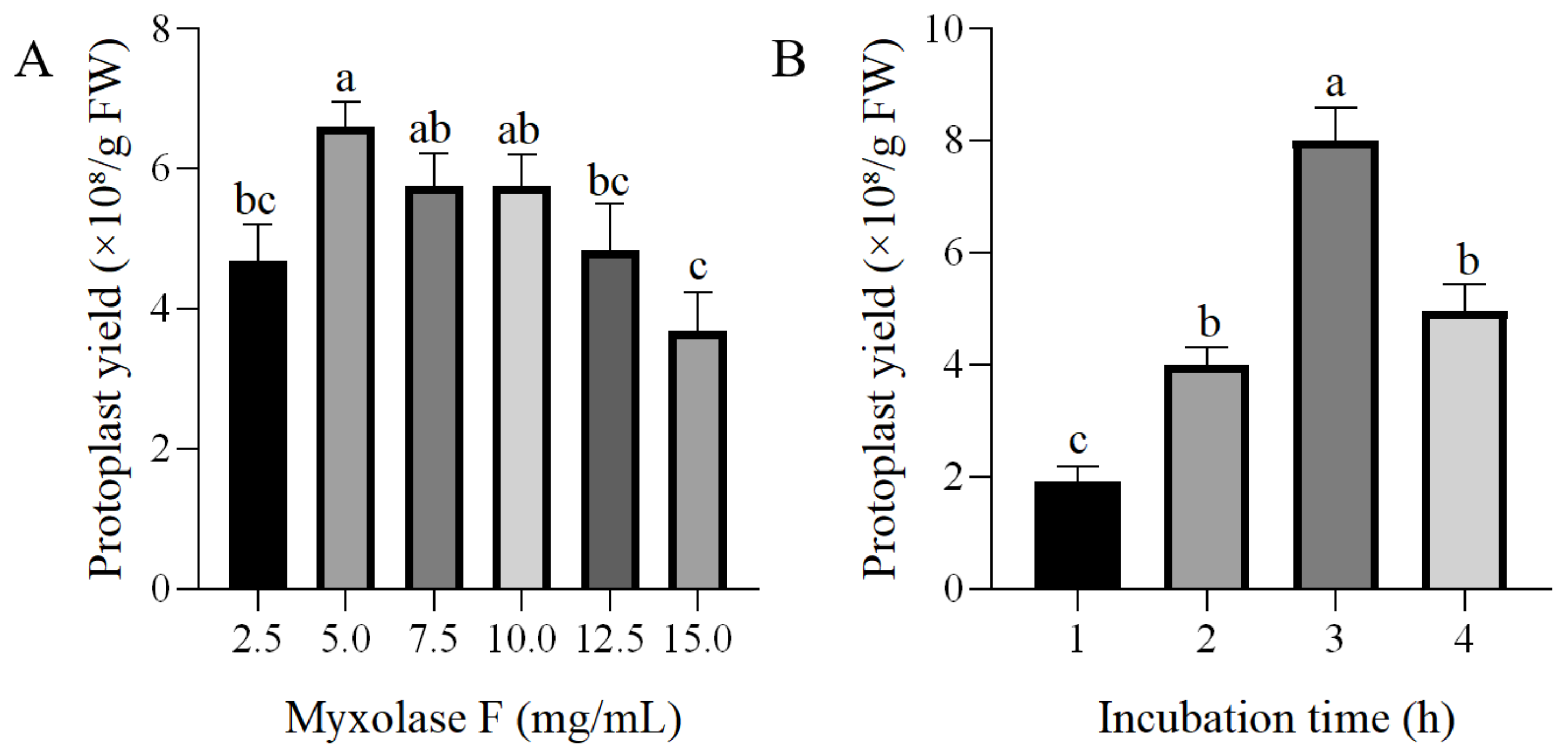
Effects of Myxolase F concentration and digestion time on the protoplast yield of *M. oryzae*. (**A**) Effect of Myxolase F concentration on protoplast yield. (**B**) Effect of digestion time on protoplast yield. Data are presented as the mean ± SD of three biological replicates. Means with different letters denote significant differences (*p* < 0.05).

**Figure 3:**
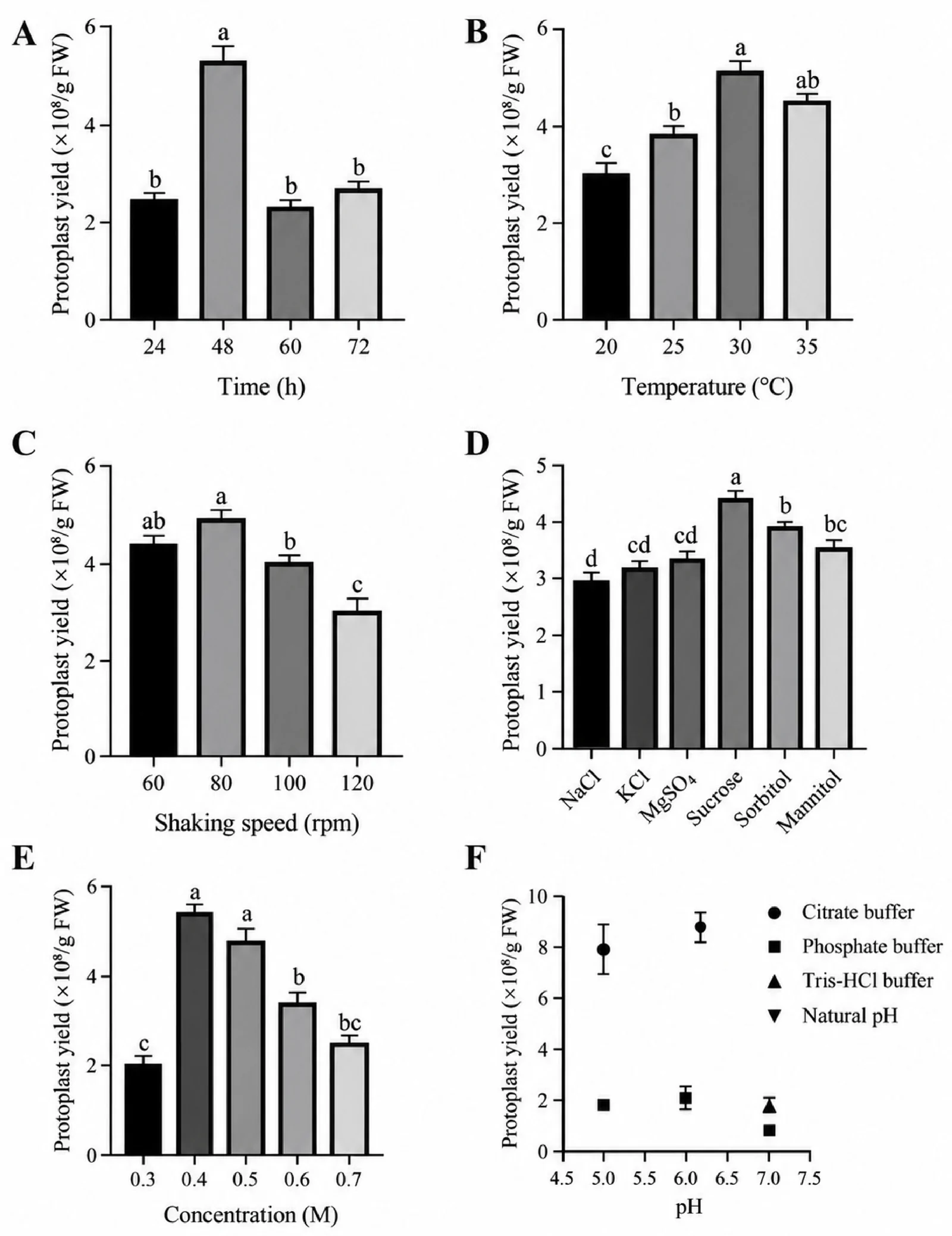
Optimization of enzymatic conditions for *M. oryzae* protoplast preparation. Effects of mycelial cultivation time (**A**), digestion temperature (**B**), shaking speed (**C**), osmotic stabilizers (**D**), sucrose concentration (**E**) and pH values (**F**) on *M. oryzae* protoplast yield following Myxolase F treatment. Data are presented as the mean ± SD of three biological replicates, and means with different letters denote significant differences (*p* < 0.05).

**Figure 4:**
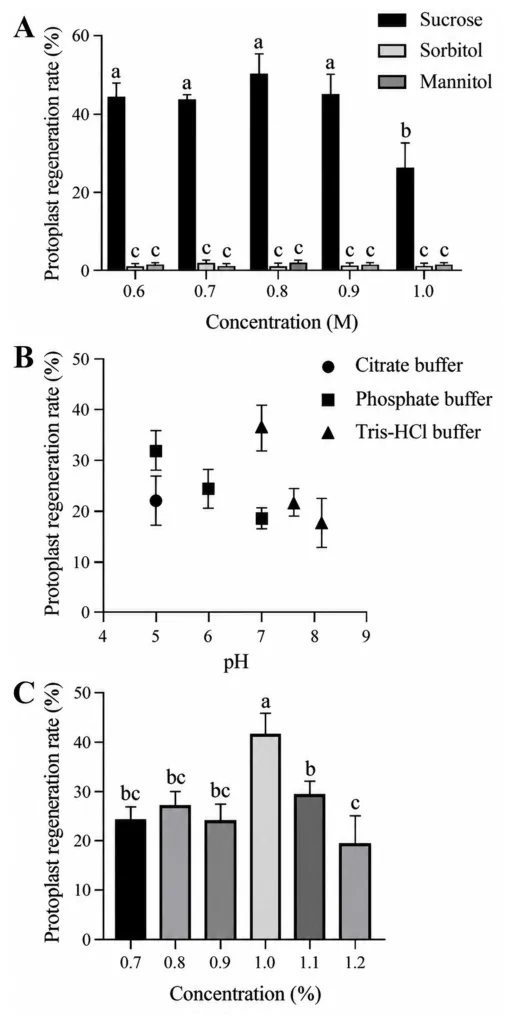
Optimization of regeneration conditions for *M. oryzae* protoplasts. (**A**) Effects of different osmotic stabilizers at various concentrations on protoplast regeneration. (**B**) Effect of pH on protoplast regeneration. (**C**) Effect of agar concentration on protoplast regeneration. Data are presented as the mean ± SD of three biological replicates, and means with different letters denote significant differences (*p* < 0.05).

**Figure 5:**
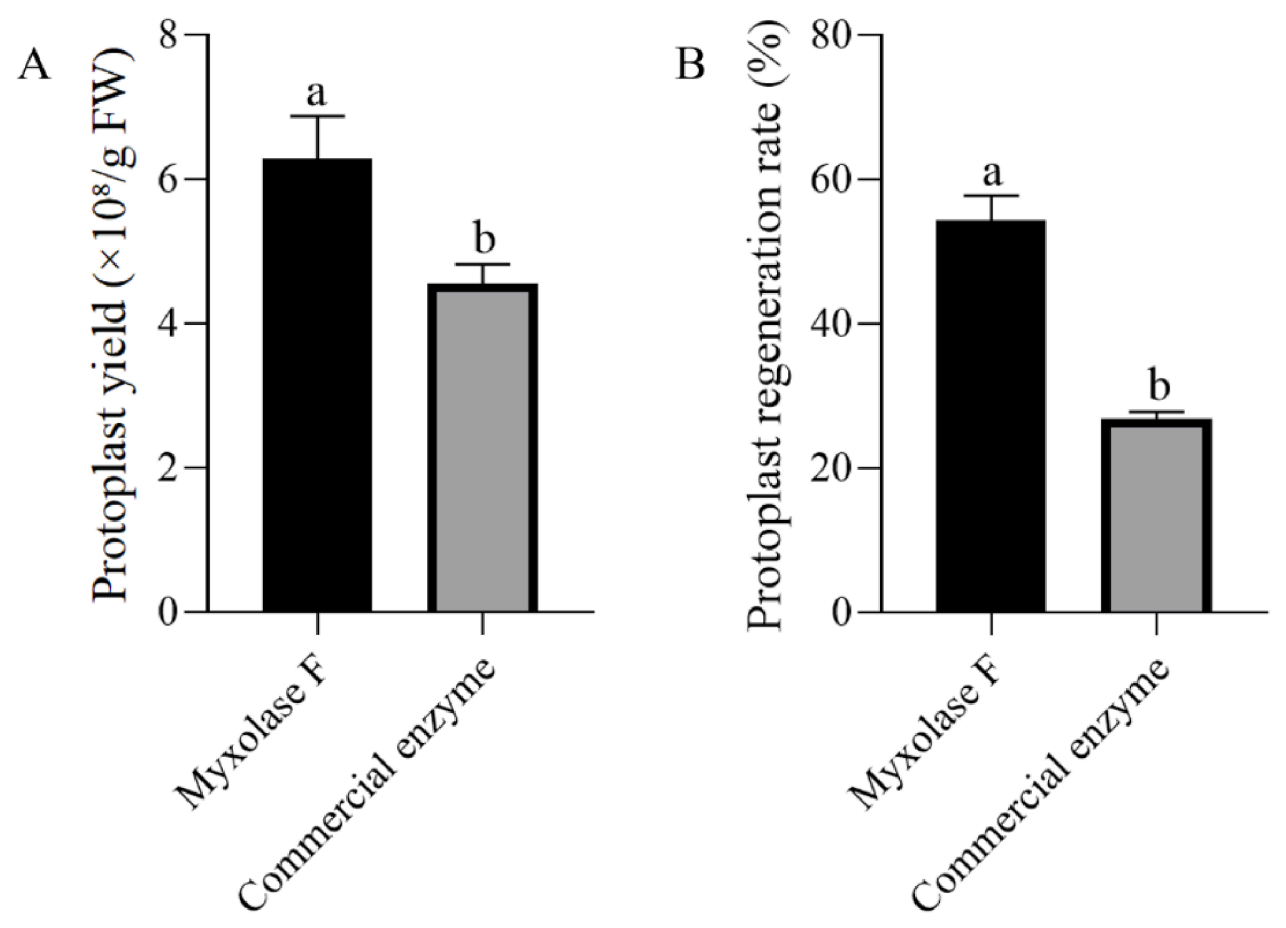
Comparison of Myxolase F with a commercial lysing enzyme in *M. oryzae* protoplast preparation and regeneration. Comparison of protoplast yield (**A**) and regeneration rate (**B**) following treatment with Myxolase F and the commercial lysing enzyme. Data are presented as the mean ± SD of three biological replicates, and means with different letters denote significant differences (*p* < 0.05).

**Figure 6:**
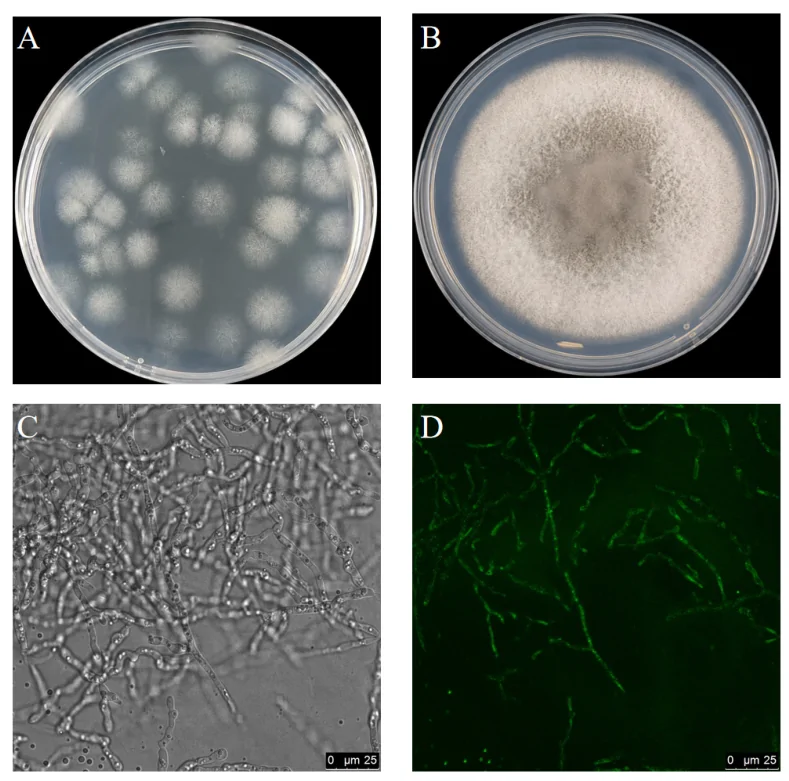
Genetic transformation in *M. oryzae* protoplasts prepared using Myxolase F. (**A**) Hygromycin B-resistant colonies obtained after PEG-mediated transformation. (**B**) Growth of selected transformants on hygromycin B-containing medium. (**C**) Bright-field image of selected transformants. (**D**) GFP fluorescence image of selected transformants. Scale bars: 25 μm.

**Table 1:** Media and solutions used for protoplast preparation and regeneration.

Medium	Standard Formula	Purpose of Use
Cm (Complete solid medium)	Dextrose 10 g, tryptone 2 g, yeast extract 1 g, casamino acids 1 g, 20× (+) NO_3_ salts 50 mL, trace elements 1 mL, vitamin solution 1 mL, and agar powder 15 g in deionized H_2_O, with the volume adjusted to 1 L.	Strain cultivation
Cm (Complete liquid medium)	CM liquid medium: dextrose 10 g, tryptone 2 g, yeast extract 1 g, casamino acids 1 g, 20× (+) NO_3_ salts 50 mL, trace elements 1 mL, and vitamin solution 1 mL in deionized H_2_O, with the volume adjusted to 1 L.	Mycelial growth
20× (+) NO_3_ salts	Sodium nitrate 120 g, potassium chloride 10.4 g, magnesium sulfate 10.4 g, and potassium phosphate 30.4 g in deionized H_2_O, with the volume adjusted to 1 L.	Component of CM medium
Trace elements	Zinc sulfate 2.2 g, boric acid 1.1 g, manganese chloride 0.5 g, iron sulfate 0.5 g, cobalt chloride hexahydrate 0.17 g, copper sulfate pentahydrate 0.16 g, sodium molybdate 0.15 g, and Na_4_EDTA 5.0 g in deionized H_2_O, with the volume adjusted to 100 mL.	Component of CM medium
Vitamin solution	Biotin 0.01 g, pyridoxine 0.01 g, thiamine 0.01 g, riboflavin 0.01 g, PABA (p-aminobenzoic acid) 0.01 g, and nicotinic acid 0.01 g in deionized H_2_O, with the volume adjusted to 100 mL.	Component of CM medium
STC	0.8 M sorbitol, 50 mM calcium chloride, and 50 mM Tris-HCl, pH 8.0.	Protoplast suspension
TB3	Yeast extract 3 g, casamino acids 3 g, agar powder 7.5 g, and sucrose 200 g in deionized H_2_O to 1 L.	Protoplast regeneration
PEG-Stabilized Tris-calcium chloride (SPTC)	5% STC and 60% (*w*/*v*) PEG 6000; PEG 6000 60 g in deionized H_2_O, with the volume adjusted to 100 mL.	Protoplast transformation
Selection medium (SM)	Yeast extract 1 g, casein enzymatic hydrolysate 1 g, agarose 10 g, and sucrose 342 g in deionized H_2_O, with the volume adjusted to 1 L.	Selection for transformed protoplast
Cm (Complete liquid medium)	CM liquid medium: dextrose 10 g, tryptone 2 g, yeast extract 1 g, casamino acids 1 g, 20× (+) NO_3_ salts 50 mL, trace elements 1 mL, and vitamin solution 1 mL in deionized H_2_O, with the volume adjusted to 1 L.	Mycelial growth

**Table 2:** Composition of TB3 buffer and osmotic stabilizers.

Buffer	Components	Adjusted pH in RM
Citric acid-NaOH	50 mM citric acid plus NaOH	5.0
KH_2_PO_4_-K_2_HPO_4_	50 mM KH_2_PO_4_ plus1M K_2_HPO_4_	5.0, 6.0 and 7.0
Tris-HCl	50 mM Tris(hydroxymethyl)aminomethane c plus HCl	7.0, 8.0 and 9.0
Glycine-NaOH	50 mM Glycine plus NaOH	9.0

## Data Availability

The raw data supporting the conclusions of this article will be made available by the authors on request.
